# Post-translational Control of RNA-Binding Proteins and Disease-Related Dysregulation

**DOI:** 10.3389/fmolb.2021.658852

**Published:** 2021-04-27

**Authors:** Alejandro Velázquez-Cruz, Blanca Baños-Jaime, Antonio Díaz-Quintana, Miguel A. De la Rosa, Irene Díaz-Moreno

**Affiliations:** Instituto de Investigaciones Químicas, Centro de Investigaciones Científicas Isla de la Cartuja, Universidad de Sevilla, Consejo Superior de Investigaciones Científicas, Seville, Spain

**Keywords:** post-translational modifications, RNA-binding proteins, liquid–liquid phase separation, HuR, TIA-1/TIAR, KSRP, hnRNP K, FUS

## Abstract

Cell signaling mechanisms modulate gene expression in response to internal and external stimuli. Cellular adaptation requires a precise and coordinated regulation of the transcription and translation processes. The post-transcriptional control of mRNA metabolism is mediated by the so-called RNA-binding proteins (RBPs), which assemble with specific transcripts forming messenger ribonucleoprotein particles of highly dynamic composition. RBPs constitute a class of *trans*-acting regulatory proteins with affinity for certain consensus elements present in mRNA molecules. However, these regulators are subjected to post-translational modifications (PTMs) that constantly adjust their activity to maintain cell homeostasis. PTMs can dramatically change the subcellular localization, the binding affinity for RNA and protein partners, and the turnover rate of RBPs. Moreover, the ability of many RBPs to undergo phase transition and/or their recruitment to previously formed membrane-less organelles, such as stress granules, is also regulated by specific PTMs. Interestingly, the dysregulation of PTMs in RBPs has been associated with the pathophysiology of many different diseases. Abnormal PTM patterns can lead to the distortion of the physiological role of RBPs due to mislocalization, loss or gain of function, and/or accelerated or disrupted degradation. This Mini Review offers a broad overview of the post-translational regulation of selected RBPs and the involvement of their dysregulation in neurodegenerative disorders, cancer and other pathologies.

## Introduction

Gene regulatory networks are constantly tuning mRNA and protein levels according to cellular needs, affecting all steps of the expression process, from transcription to protein degradation, and including mRNA maturation, transport and translation (Adeli, 2011). mRNA molecules are permanently associated with a dynamic set of proteins and non-coding RNAs, such as microRNAs (miRNAs), in the so-called messenger ribonucleoprotein particles (mRNPs). RNA-binding proteins (RBPs) serve different purposes within mRNPs and their combined effects determine the fate of the transcript at each stage of its life cycle (Müller-McNicoll and Neugebauer, 2013; Mitchell and Parker, 2014).

The interaction of most known RBPs with their cognate transcripts is mediated by a small group of RNA-binding domains (RBDs), such as the RNA recognition motif (RRM), K homology (KH), zinc-finger and DEAD/DEAH box helicase. These structural motifs have been extensively studied, their modes of interaction are widely known and represent a criterion for the classification of RBPs (Corley et al., 2020). RBPs usually contain several repeats of the same RBD, which synergistically contribute to improve the specificity and affinity for their target mRNAs (Gerstberger et al., 2014; Corley et al., 2020). However, an increasing number of RBPs lacks a defined or ‘classic’ RBD, harboring instead at least one intrinsically disordered region (IDR) through which they can bind to RNA with a wide spectrum of affinities (Hentze et al., 2018).

### Control of mRNA Life Cycle

RBPs constitute a class of *trans*-acting regulatory proteins with affinity for certain consensus sequences present in RNA molecules. Most of the identified and well-studied RBPs specifically bind mRNA, typically through the recognition of *cis*-acting elements located in the 5′ and 3′ untranslated regions (UTRs), although binding sites can also be found in the coding sequence (Gerstberger et al., 2014; Hentze et al., 2018; Van Nostrand et al., 2020). Since a particular *cis*-acting sequence is typically shared by many different transcripts, a single RBP can control the expression of multiple mRNAs and can thus profoundly alter cellular functions (Keene, 2010; Adeli, 2011; Müller-McNicoll and Neugebauer, 2013). In fact, there are many functionally related mRNAs that display a common element for co-regulation by RBPs, which is essential for a rapid and coordinated response to physiological and stress signals (Keene, 2010; Pope and Medzhitov, 2018). In this regard, one of the most important advantages of RNA networks is their great versatility, characterized by a constantly fluctuating transcriptome, thanks to the ability of the mRNA synthesis and degradation machinery to operate at a relatively high pace (Keene, 2010).

Numerous RBPs can associate with the same mRNA, either cooperating or competing for binding (Rougemaille et al., 2008; Müller-McNicoll and Neugebauer, 2013; Mitchell and Parker, 2014; Van Nostrand et al., 2020). The interaction with RBPs influences the maturation of mRNAs (alternative polyadenylation and splicing) and their cellular distribution, and either increases or decreases their stability, translation and degradation (Witten and Ule, 2011; Erson-Bensan, 2016; García-Mauriño et al., 2017). Many RBPs can also bind to their own transcript and/or that of other RBPs. The cross-talk between RBPs is an essential part of the regulation of this class of proteins and, therefore, of the gene expression itself (García-Mauriño et al., 2017).

The biosynthesis and function of miRNAs are also regulated by RBPs, with relevant consequences for mRNA fate. In the canonical miRNA biogenesis pathway, immature miRNAs are typically transcribed into long primary transcripts (pri-miRNAs) bearing a stem-loop structure. These pri-mRNAs are cleaved in the nucleus by the ribonuclease Drosha, which is part of the so-called microprocessor complex, along with two molecules of the RBP termed DiGeorge syndrome critical region gene 8 (DGCR8). The resulting pre-miRNAs are translocated by the exportin-5 receptor to the cytoplasm, where another RNAse, Dicer, recognizes and cleaves their hairpin motif producing miRNA duplexes. Then, the Argonaute (AGO) proteins bind to double-stranded miRNAs and both assemble into the miRNA-induced silencing complex (miRISC), where one strand of the mRNA duplex becomes functional and the other is removed. Complementary base pairing of the mature single-stranded miRNA with sequences of target mRNAs, mostly located in the 3′ UTRs, guides the translational inhibition and/or RNA degradation activity of miRISC (Zealy et al., 2017; Correia de Sousa et al., 2019; Michlewski and Cáceres, 2019).

RBPs can both up- and downregulate miRNAs at various levels, through direct binding to pri-/pre-miRNAs and/or indirectly via interaction with components of the miRNA processing machinery and their transcripts (e.g., modifying the expression and activity of Drosha and Dicer, or miRNA loading into miRISC) (Iadevaia and Gerber, 2015; Loffreda et al., 2015). Moreover, RBPs can either facilitate or prevent miRNAs binding to mRNAs, thus modulating their translational repression activity (Iadevaia and Gerber, 2015).

### Intrinsic Phase Separation Ability

RBPs are major constituents of membrane-less organelles (MLOs) or condensates, which are dynamic macromolecular assemblies that become segregated from the surrounding protoplasm through the process of liquid-liquid phase separation (LLPS) (Owen and Shewmaker, 2019; Ryan and Fawzi, 2019). The nucleoplasm and cytosol are not homogeneous fluids, but instead contain different liquid-droplet phases where proteins and/or RNA are accumulated (Brangwynne, 2013; Ryan and Fawzi, 2019). Once certain concentration threshold is reached, these biomolecules assemble into different MLOs with a specific composition and physiological function (Darling et al., 2018). For example, the cytosolic stress granules (SGs)—which are formed in response to diverse stimuli—typically include the RBPs T-cell intracellular antigen 1 (TIA-1) and poly(A)-binding protein (PABP) and are associated to mRNA metabolism (Darling et al., 2018; Wolozin and Ivanov, 2019). Other MLOs, constantly present under homeostasis, are nucleoli, which are distinctively enriched in the RBP nucleolin (NCL) and specialized in ribosome biogenesis (Lo et al., 2006; Darling et al., 2018).

Due to the relatively weak, non-covalent nature of the interactions stablished by MLO components, diffusion of biomolecules into and out of the condensates is favored, which allows the fast assembly and disassembly of these structures (Brangwynne, 2013; Drino and Schaefer, 2018). The total interaction strength provided by the network of multivalent contacts between proteins and/or RNA is high enough to promote LLPS while ensuring reversible associations and great mobility inside condensates (Strom and Brangwynne, 2019). RBPs can contribute to phase separation by using both well-structured RBDs and IDRs for binding with RNA molecules, which act as scaffolds during condensation and determine the physicochemical and material properties of the resulting MLO (Drino and Schaefer, 2018; Ryan and Fawzi, 2019; Loughlin et al., 2021). IDRs from RBPs are also involved in protein-protein interactions, including self-association, thanks to the high proportion and distribution pattern of particular residues in their sequences, often clustered in repetitive low-complexity regions (LCRs), such as the arginine/glycine-rich (RGG/RG) boxes and the glutamine/asparagine-rich prion-like domains (PLDs) (Darling et al., 2018; Ryan and Fawzi, 2019). The weak cation–π intermolecular interactions between arginines from RGG motifs and aromatic residues (mostly tyrosines) of RBPs—e.g., heterogeneous nuclear ribonucleoprotein A2 (hnRNP A2) and fused in sarcoma (FUS)—are the driving force for their aggregation and liquid demixing (Hofweber et al., 2018; Qamar et al., 2018; Ryan et al., 2018; Hofweber and Dormann, 2019).

MLOs play an essential role in the cell by enabling the controlled and selective concentration of particular RBPs, among other biomolecules, to carry out critical biochemical reactions under optimal conditions, separated from the rest of their environment (Drino and Schaefer, 2018; Strom and Brangwynne, 2019). However, dysregulation of phase-separating RBPs such as FUS and TAR DNA-binding protein of 43 KDa (TDP-43) can lead to irreversible and aberrant condensate formation, a process deleterious to cells and frequently associated with neurodegenerative diseases, as discussed below (Bowden and Dormann, 2016; St George-Hyslop et al., 2018; Fernandopulle et al., 2019; Xue et al., 2020).

### PTM-Dependent Activity

As noted previously, RBPs are prominently involved in the post-transcriptional modulation of mRNAs, accompanying them throughout their entire life-cycle. Nonetheless, the activity of RBPs is, in turn, heavily controlled by post-translational modifications (PTMs), which constitute an extra layer of regulation of gene expression. PTMs of proteins refer to generally enzymatic reactions that occur after their synthesis and consist of the covalent addition of small functional groups (e.g., phosphate, methyl and acetyl) or biomolecules (e.g., peptides, glycans and lipids) to one amino acid, its chemical modification (e.g., citrullination) and the cleavage of peptide bonds (e.g., caspase proteolysis) (Lovci et al., 2016; Virág et al., 2020). PTMs can dramatically change the properties of RBPs, including subcellular localization, association with target RNAs and other RNA-associated proteins, and degradation. This Mini Review focuses on the role of important PTMs (listed in Supplementary Table 1) in the biology of a subset of well-studied RBPs (listed in Supplementary Table 2), and their relevance in the development of various diseases. To this end, multiple examples are provided, which highlight the profound effects that these changes produce in RBPs under different cellular contexts. Note that the comprehensive compilation of the totality of PTMs described for all identified RBPs is beyond the scope of this Mini Review. All selected examples, including proteins, chemical modifications and diseases, are intended to qualitatively represent the intricate mechanisms and medical implications underlying the post-translational regulation and dysregulation of RBPs.

## Regulation of RBP Biology by PTMs

A rigorous, yet dynamic regulation of PTMs on the entire population of RBPs is essential for the maintenance of cellular balance, since these chemical marks have the potential to reconfigure the structure and redefine the function of RBPs. In the following sections, we will delve into the main characteristics of RBPs subject to significant variations due to PTMs.

### Subcellular Localization

There are multiple reports on the influence that PTMs exert on the subcellular distribution of RBPs, with important consequences on RNA metabolism, either due to impaired nuclear export or spatial separation from their target RNAs into different compartments. For example, many of the PTMs identified in the RBP human antigen R (HuR) alter its localization. Most are phosphorylations and a great part of the modified residues are present in the so-called hinge region, an unstructured stretch containing the HuR nucleocytoplasmic shuttling (HNS) sequence (Grammatikakis et al., 2017). These phosphorylations are associated with the cytosolic accumulation of HuR, normally triggered by stress, since this RBP is predominantly nuclear in unstimulated cells (Doller et al., 2007, 2008, 2010; Kim et al., 2008; Lafarga et al., 2009; Chu et al., 2012; Filippova et al., 2012; Yoon et al., 2014).

Subcellular localization of the serine/arginine-rich (SR) protein family of splicing factors is also regulated by phosphorylations. Mostly nuclear, some members such as serine/arginine-rich splicing factor 1 (SRSF1) and SRSF3 can shuttle to the to participate in other post-transcriptional processes. However, the nuclear import of these RBPs through the transportin (TRN)-SR2 requires phosphorylation by the SR protein kinases 1 and 2 (SRPK1/2) (Lai et al., 2000, 2001; Long et al., 2019). In contrast, another previously identified importin-β, TRN-SR1, mediates the nuclear translocation of unphosphorylated SR proteins (Kataoka et al., 1999), although it remains unclear whether it can also transport phosphorylated forms (Kataoka et al., 1999; Lai et al., 2000, 2001).

The localization of another well-known RBP, hnRNP K, relies on several phosphorylatable serines and methylatable arginines (Xu et al., 2019). In general, phosphorylations at the K-interactive region (KI) and the K-nuclear shuttling domain (KNS) of hnRNP K control its subcellular distribution. For instance, this primarily nuclear RBP was shown to increase its cytosolic levels after phosphorylation by extracellular signal-regulated kinase (ERK) at Ser284 and Ser353 (Habelhah et al., 2001a,b; Huang et al., 2017). However, a phosphoproteomic study of hnRNP K revealed that phospho-Ser116 could also be involved in the subcellular distribution of this RBP. Furthermore, the same study linked the phosphorylation of Ser284 to the nuclear accumulation of hnRNP K (Kimura et al., 2010), in contrast to other reports (Habelhah et al., 2001a,b; Huang et al., 2017). On the other hand, methylations at the intrinsically disordered RGG-box of hnRNP K by the arginine N-methyltransferase 1 (PRMT1) have been related to the nuclear retention of this RBP (Chang et al., 2011).

**FIGURE 1:**
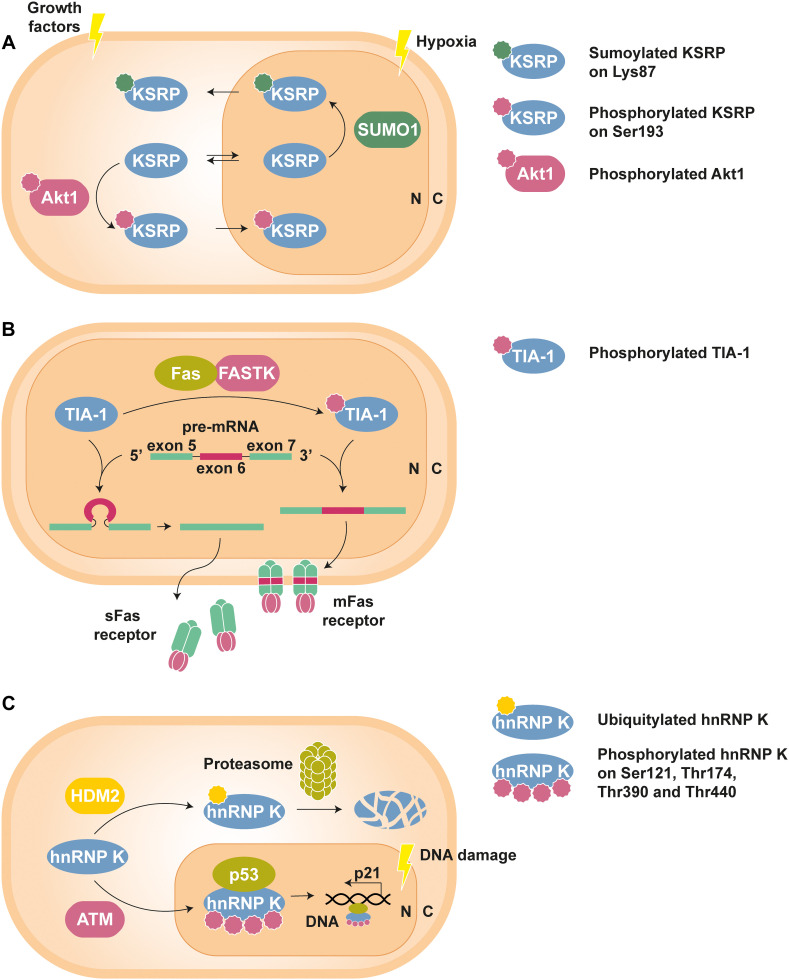
Examples of PTM-mediated regulation of RBPs. **(A)** KSRP can shuttle between nucleus (N) and cytoplasm (C) to perform specific functions in each compartment. However, phosphorylation at Ser193 by Akt1, stimulated by growth factors, promotes the translocation of KSRP to the nucleus, whereas hypoxia-induced SUMOylation at Lys83 leads to its nuclear export (Díaz-Moreno et al., 2009; Yuan et al., 2017). **(B)** Phosphorylation of TIA-1 by FASTK improves its ability to recruit the U1 snRNP spliceosomal complex to the 5′ splice site region of the Fas receptor pre-mRNA exon 6. The resulting mature mRNA will express mFas, which plays an important role in the extrinsic apoptosis signaling pathways. In contrast, splicing of Fas receptor in the presence of unphosphorylated TIA-1 results in exon 6 skipping and the synthesis of sFas, that blocks apoptosis (Förch et al., 2002; Izquierdo and Valcárcel, 2007). **(C)** Under standard conditions, hnRNP K is targeted by the E3 Ub-ligase HDM2 for proteasomal degradation. Nonetheless, DNA damage triggers ATM-dependent phosphorylation of hnRNP K at Ser121, Thr174, Thr390, and Thr440, thus lowering its turnover rate. In addition, phosphorylated hnRNP K stimulates p53-mediated p21 gene expression, which causes cell cycle arrest (Moumen et al., 2005, 2013).

An example of interplay between PTMs can be found in the nucleo-cytoplasmic shuttling of the KH-type splicing regulatory protein (KSRP). Phosphorylation of this RBP at Ser193 by Akt1 causes the unfolding of its first KH domain, giving rise to a binding site for the chaperone protein 14-3-3ζ, whose interaction is involved in the nuclear confinement observed for the phospho-isoform of KSRP (Díaz-Moreno et al., 2009). On the contrary, SUMOylation, i.e., the covalent attachment of the small ubiquitin-like modifier (SUMO) peptide, of KSRP at Lys87 by SUMO1 promotes nuclear export and increases its cytosolic levels (Figure 1A) (Yuan et al., 2017).

A less known PTM called myristoylation, i.e., the covalent addition of the fatty acid myristate to a N-terminal glycine, controls the axonal distribution of the neuronal fragile X-related protein 2 (FXR2P), restricting the localization of this RBP to proximal axon segments (Stackpole et al., 2014).

There are many documented cases of PTMs regulating the compartmentalization of RBPs into diverse MLOs. For instance, FUS acetylation at Lys510 by CREB-binding protein (CBP)/p300 impedes its nuclear import via TRN1, sequestering this RBP in the cytoplasm where it assembles into stress granule-like inclusions (Arenas et al., 2020). The opposite occurs to HuR when it is phosphorylated at Tyr200 by Janus kinase 3 (JAK3), as this PTM prevents its localization in arsenite-induced SGs (Yoon et al., 2014). Similar to HuR, Ras-GAP SH3 domain-binding protein 1 (G3BP1) phosphorylation at Ser149 might regulate its ability to mediate SG assembly (Tourrière et al., 2003). However, a recent examination of this hypothesis attributes the observed phenotype to an accidental mutation in the G3BP1 S149E phosphomimetic construct (Panas et al., 2019).

As another example, acetylation of NCL at Lys88 can mobilize this nucleolar RBP to the nuclear speckles, one type of MLO enriched in splicing and transcription factors, suggesting the involvement of NCL in mRNA synthesis and processing (Das et al., 2013). Conversely, phosphorylation by Cdc2-like kinase 1 (Clk1) facilitates the release of SRSF1 from nuclear speckles to the nucleoplasm (Ngo et al., 2005).

### Interactions With Transcripts and Other RNA-Associated Proteins

PTMs can either facilitate or hinder the interaction of RBPs with their cognate transcripts and other RNA-associated proteins. For example, HuR binds to AU-rich elements (ARE) in the 3′ UTRs of target mRNAs (ARE-RBP), normally stabilizing them and/or enhancing their translation (García-Mauriño et al., 2017; Pabis et al., 2019). Phosphorylation of this ARE-RBP at residues within or near its three RRM domains often modulates the interaction with transcripts (Grammatikakis et al., 2017). For example, the ionizing radiation-triggered phosphorylation at Ser88, Ser100 and Thr118 by the checkpoint kinase 2 (Chk2) disengages HuR from most mRNA complexes throughout the cell, favoring its survival (Masuda et al., 2011). Strikingly, a previous report also associated the aforementioned Chk2-mediated phosphorylations on HuR, especially at Ser100, with impaired binding to Sirtuin 1 (SIRT1) mRNA but higher levels of cell death under oxidative stress conditions (Abdelmohsen et al., 2007). On the contrary, there are other documented cases involving Ser88- (Yu et al., 2011) and Ser100-phosphorylation (Liu et al., 2009) by Chk2, in which an increased binding of HuR to a specific mRNA was observed. Indeed, many phosphorylations by other kinases such as p38 mitogen-activated protein kinase (MAPK) and protein kinase C α and δ (PKCα/δ) lead to higher HuR affinity for certain transcripts (Doller et al., 2007, 2010; Lafarga et al., 2009; Bergalet et al., 2011; Liao et al., 2011; Gummadi et al., 2012; Scheiba et al., 2012).

Alternative PTMs can also modulate the interaction between HuR and mRNA with antagonistic effects. For instance, Arg217-methylation by coactivator-associated arginine methyltransferase 1 (CARM1) promotes HuR association with transcripts (Li et al., 2002; Calvanese et al., 2010; Pang et al., 2013), whereas ubiquitylation by the ubiquitin regulatory X domain-containing protein 8 (UBXD8)-p97 complex dissociates HuR from mRNPs (Zhou et al., 2013).

hnRNP K preferentially interacts with CU-rich elements (CUREs), such as the differentiation control element (DICE), in the 3′ UTRs of mRNAs and undergoes phosphorylations at residues within or near the three KH domains that regulate its association with nucleic acids (Xu et al., 2019). For example, Tyr458-phosphorylation by Src kinase impairs the KH3-mediated binding of hnRNP K to the transcripts of erythroid-15-lipoxygenase (r15-LOX) (Ostareck-Lederer et al., 2002; Messias et al., 2006) and uncoupling protein-2 (UCP2) (Tahir et al., 2014), thus suppressing the inhibitory effect on translation by this RBP. Moreover, the KH3 domain of hnRNP K is removed by caspase-3 cleavage at Asp334, ‘unlocking’ r15-LOX mRNA translation during erythroid differentiation (Naarmann-de Vries et al., 2013). Interestingly, arginine methylation of hnRNP K RGG motif by PRMT1 precludes its phosphorylation by Src (Ostareck-Lederer et al., 2006) and PKCδ (Yang et al., 2014), which have important repercussions for the DNA damage response (DDR). Under genotoxic stress, methylated hnRNP K shows an increased affinity for the apoptosis regulator p53 and enhances its transcriptional activity, thus facilitating cell cycle arrest and DNA repair (Chen et al., 2008).

AU-rich binding factor 1 (AUF-1) generally promotes mRNA decay and possesses four splice isoforms (García-Mauriño et al., 2017). The p40 isoform (p40^AUF1^) is phosphorylated at Ser83 and Ser87 by glycogen synthase kinase 3β (GSK3β) and protein kinase A (PKA), respectively. p40^AUF1^ form dimers that bind sequentially to the ARE sequence from the tumor necrosis factor α (TNFα) mRNA, up to a maximum of two dimers per RNA oligo. *In vitro* binding assays showed that single Ser83-phosphorylation reduces by roughly 40% the binding of p40^AUF1^ dimers to a free TNFα-ARE oligo. Intriguingly, when p40^AUF1^ dimers are singly-phosphorylated at Ser87, the affinity of the second binding event increases twice, relative to the unphosphorylated p40^AUF1^ dimer. Moreover, simultaneous phosphorylation of both serines has the same impact on p40^AUF1^ interaction with TNFα-ARE oligo than Ser83 single-phosphorylation, i.e., the negative effect of phospho-Ser83 dominates the positive one by phospho-Ser87 (Wilson et al., 2003).

The function of KSRP and AUF-1 as mediators of exosomal mRNA decay is regulated by the protein interacting with NIMA (never in mitosis A)-1 (Pin1) enzyme, which specifically isomerizes phosphorylated Ser/Thr-Pro peptide bonds (Shen and Malter, 2015). Phospho-isoforms of these ARE-RBPs are modified by Pin1, impacting on their affinity for mRNA. For example, prolyl isomerization increases the binding of KSRP to the parathyroid hormone (PTH) mRNA (Nechama et al., 2009) while attenuates the association of all four AUF-1 isoforms with the granulocyte-macrophage colony-stimulating factor (GM-CSF) mRNA (Shen et al., 2005). Of note, Pin1 activity plays a prominent role in the inflammatory and immune response, as several RBP substrates of this enzyme control the expression of many cytokines (Shen and Malter, 2015).

Tristetraprolin (TTP) is another ARE-RBP that facilitates mRNA degradation, including its own transcript (García-Mauriño et al., 2017). Phosphorylation of TTP at Ser52 and Ser178 by MAPK-activated protein kinase 2 and 3 (MAPKAPK-2/3 or MK2/3) improves its stability and expression, but also diminishes its capacity to recruit deadenylases to target mRNAs, among which are the transcripts of many cytokines (Hitti et al., 2006; Ronkina et al., 2007, 2019; Clement et al., 2011). In fact, there is much evidence pointing to an essential role of TTP phosphorylation and dephosphorylation in the regulation of inflammation (Clark and Dean, 2016). Ser52- and Ser178-phosphorylation is necessary and sufficient to suppress the mRNA-destabilizing activity of TTP through complex formation with the 14-3-3 chaperone, allowing the expression and participation of its target cytokines in the inflammatory response, until reactivation of TTP via dephosphorylation. However, MK2/3 phosphorylates several other residues of TTP and it has been proposed, based on recent experimental data, that phospho-Ser316 could contribute to the complete inactivation of TTP (Ronkina et al., 2019).

Structural details on the binding of TIA-1 to RNA have been thoroughly determined (Aroca et al., 2011; Bauer et al., 2012; Cruz-Gallardo et al., 2013, 2014, 2015; Wang et al., 2014; Waris et al., 2017; Loughlin et al., 2021), although very little information is available about the effect of PTMs on this ARE-RBP. Nevertheless, it is well-known that phosphorylation of TIA-1 and its homolog TIA-1-related protein (TIAR) modulates their activity in the alternative splicing of the Fas receptor. Pre-mRNA splicing radically influences the properties and function of the Fas receptor expressed by the mature mRNA: the inclusion of exon 6 gives rise to a pro-apoptotic membrane protein (mFas), whereas the skipping of this exon determines the synthesis of a soluble (sFas) and anti-apoptotic isoform (Ruberti et al., 1996). TIA-1/TIAR phosphorylation by FASTK has been associated to the expression of the mFas isoform (Figure 1B). Furthermore, TIA-1 phosphorylation by FASTK has been shown to increase the recruitment of the spliceosomal U1 small nuclear RNP (snRNP U1) to Fas receptor pre-mRNA suboptimal 5′ splice sites (Izquierdo and Valcárcel, 2007), presumably due to enhanced interaction of phospho-TIA-1 with the U1-C protein subunit (Förch et al., 2002). Importantly, the binding affinity of TIA-1 for RNA remained unaltered upon phosphorylation (Izquierdo and Valcárcel, 2007).

PTM-mediated disruption of the complexes between phase-separating RBPs and RNA can alter their condensation capacity. Such is the case for the acetylation of FUS (Lys315 and Lys316) and G3BP1 (Lys376) by CBP/p300, which impairs the binding of these RBPs to target RNAs (Gal et al., 2019; Arenas et al., 2020). Moreover, it has been proposed that lysine acetylation of G3BP1 assists SGs disassembly, a physiological mechanism that could be exploited for therapeutic purposes (Gal et al., 2019).

### Turnover and Degradation

Maintenance of cell homeostasis requires tight regulation of protein concentrations (Hanna et al., 2019). Protein turnover, i.e., the dynamic balance between synthesis and degradation, ensures the replacement of old proteins, potentially defective and/or harmful, with new copies, and the adaptation of the proteome composition to different cellular contexts and stimuli (Toyama and Hetzer, 2013; Alber and Suter, 2019).

There are two main pathways for protein degradation: the autophagy lysosomal pathway (ALP) and the ubiquitin (Ub)-proteasome system (UPS). The ALP consists of the unspecific breakdown of cellular material (including non-protein biomolecules and even complete organelles), which is isolated in double-membrane vesicles called autophagosomes and digested after lysosome fusion. In contrast, the UPS is based on the labeling of proteins with the 76-amino acid polypeptide Ub for their targeting and destruction by large protease complexes termed proteasomes (Ohsumi, 2006; Varshavsky, 2017). The covalent binding of Ub to lysine residues requires the concerted action of three enzymes generically known as E1, E2 and E3 (Alber and Suter, 2019). Susceptible proteins carry degradation signals or ‘degrons’ that allow their recognition and binding by E3 Ub-protein ligases (Geffen et al., 2016). Then, targeted proteins can be mono-ubiquitylated on one or more lysines (multi-mono-ubiquitylation), and poly-ubiquitylated, i.e., attached to a poly-Ub chain of a variable length and structure. Finally, ubiquitylated proteins are processed by the proteasomal machinery and their components are subsequently recycled (Hanna et al., 2019).

The abovementioned three-tier process is also used for the attachment of Ub-like proteins (e.g., SUMO and neural precursor cell expressed developmentally downregulated 8 or NEDD8) to target substrates (Enchev et al., 2015; Pichler et al., 2017). Importantly, ubiquitylation, SUMOylation and NEDDylation are involved in different cellular events other than degradation, as shown by various examples throughout this Mini Review.

The cellular levels of RBPs, as master regulators of gene expression, are continuously adjusted via UPS. For instance, KSRP proteasome-mediated turnover controls the exosome recruitment activity of this ARE-RBP for target mRNA degradation (García-Mayoral et al., 2007; Díaz-Moreno et al., 2010; Gherzi et al., 2010; Briata et al., 2013; Wang et al., 2020). As another example, HuR ubiquitylation specifically at Lys182 has been related to a decrease in its cellular levels after heat shock (Abdelmohsen et al., 2009).

Crosstalk between PTMs targeting the same RBP has a decisive influence on its turnover rate. HuR phosphorylation at Ser304 and Ser318 by IκB kinase α (IKKα) and PKCα, respectively, precedes its ubiquitylation and subsequent degradation in cancer cells upon glycolysis inhibition. Specifically, Ser318-phosphorylation facilitates HuR nuclear export, whereas phospho-Ser304 is essential for HuR binding to the E3 Ub-ligase β-transducin repeat-containing protein 1 (β-TrCP1). Interestingly, β-TrCP1 recognizes a particular sequence stretch in HuR RRM3, which includes Ser304 at its N-terminal end (Chu et al., 2012).

Similarly, the phosphorylation of the molecular chaperone and RBP heat shock protein 27 (Hsp27) drives AUF-1 proteolysis and indirectly increases the half-life of ARE-containing mRNAs. Hsp27 Ser15-phosphorylation by p38 MAPK and/or MK2 could trigger its proteasomal co-degradation with AUF-1, thus preventing the destabilizing effect of both ARE-RBPs (Knapinska et al., 2011). In contrast, the previously mentioned MK2/3-mediated phosphorylations that inactivate TTP, i.e., phospho-Ser52 and phospho-Ser178, also protect it against proteasomal degradation. However, it has been described that TTP can be processed by the proteasome in a Ub-independent manner through degradation ‘by default,’ in which the presence of IDRs in the RBP would be essential (Ngoc et al., 2014).

The expression and turnover rate of the splicing factors SRSF2 and hnRNP A1 are significantly affected by the action of α-ketoglutarate-dependent hydroxylases. Prolyl hydroxylation of these RBPs lowers their degradation rate, although it also downregulates hnRNP A1 protein synthesis (Stoehr et al., 2016).

The role of hnRNP K in DDR is also regulated by protein turnover. The E3 Ub-ligase human/mouse double minute 2 (HDM2/MDM2) targets hnRNP K for proteasomal degradation in undamaged cells. Nevertheless, genotoxic stimuli trigger ataxia-telangiectasia mutated (ATM)-mediated phosphorylation of hnRNP K at Ser121, Thr174, Thr390, and Thr440, with the consequent decrease in the turnover of the RBP and increase in its activity as p53 transcriptional co-activator (Figure 1C) (Moumen et al., 2005, 2013). DNA damage was also shown to induce hnRNP K SUMOylation at Lys422 by the E3 SUMO-ligase polycomb 2 (Pc2), thereby leading to upregulation of p53 function (Pelisch et al., 2012).

## PTMs of RBPs in the Pathophysiology of Diseases

Given the extraordinary relevance of RBPs for the viability and correct functioning of the cell, it is not surprising that their dysregulation is involved in the etiology and/or pathogenesis of a wide variety of diseases (Vidal, 2011; Gerstberger et al., 2014; Conlon and Manley, 2017; Pereira et al., 2017; Moore et al., 2018; Gebauer et al., 2021). Next, the molecular mechanisms that connect some PTMs of RBPs with various pathologies are described, with special attention to neurodegenerative diseases and cancers.

### Neurodegenerative Diseases

The presence of intracellular protein aggregates is pathognomonic for neurological disorders such as fronto-temporal lobar degeneration (FTLD) and amyotrophic lateral sclerosis (ALS). Besides the classic β-amyloid deposits, the abnormal accumulation of various RBPs within gel-like or insoluble droplets in neurons and glia has been widely documented and their study has aroused increasing interest during the last decade. The irreversible solidification experienced by condensates is often associated with key mutations in RBP-encoding genes. However, the ability of both wild-type and mutant RBPs to undergo LLPS and gelation can be profoundly altered by PTMs (Bowden and Dormann, 2016; St George-Hyslop et al., 2018; Fernandopulle et al., 2019; Xue et al., 2020). For example, aberrant hyper-phosphorylation, ubiquitylation, acetylation, cysteine oxidation and caspase cleavage of TDP-43 have been related to a pathogenic behavior of this RBP in FTLD and/or ALS, including loss of physiological function, mislocalization and higher aggregation (Buratti, 2018; François-Moutal et al., 2019; Prasad et al., 2019).

PARylation, i.e., the covalent addition of poly(ADP-ribose) or PAR residues, is another important PTM for the phase separation of the RBPs TDP-43 and hnRNP A1. TDP-43 binding to PAR molecules stimulates the recruitment of this RBP to SGs, temporarily blocking its pathological phosphorylation. However, tankyrase-1/2 PARylation activity under chronic stress has been correlated to increased accumulation of phosphorylated TDP-43 in cytosolic foci, which may eventually evolve to a solid-like state (McGurk et al., 2018). On the other hand, Lys298-PARylation of hnRNP A1 by PAR polymerase 1 (PARP-1) is involved in stress-induced cytosolic translocation, while the PAR-binding ability of hnRNP A1 positively controls its association and co-LLPS with TDP-43 (Duan et al., 2019). Interestingly, toxicity of both PAR ‘readers’ is proportional to the cellular PARylation levels (McGurk et al., 2018; Duan et al., 2019).

**FIGURE 2:**
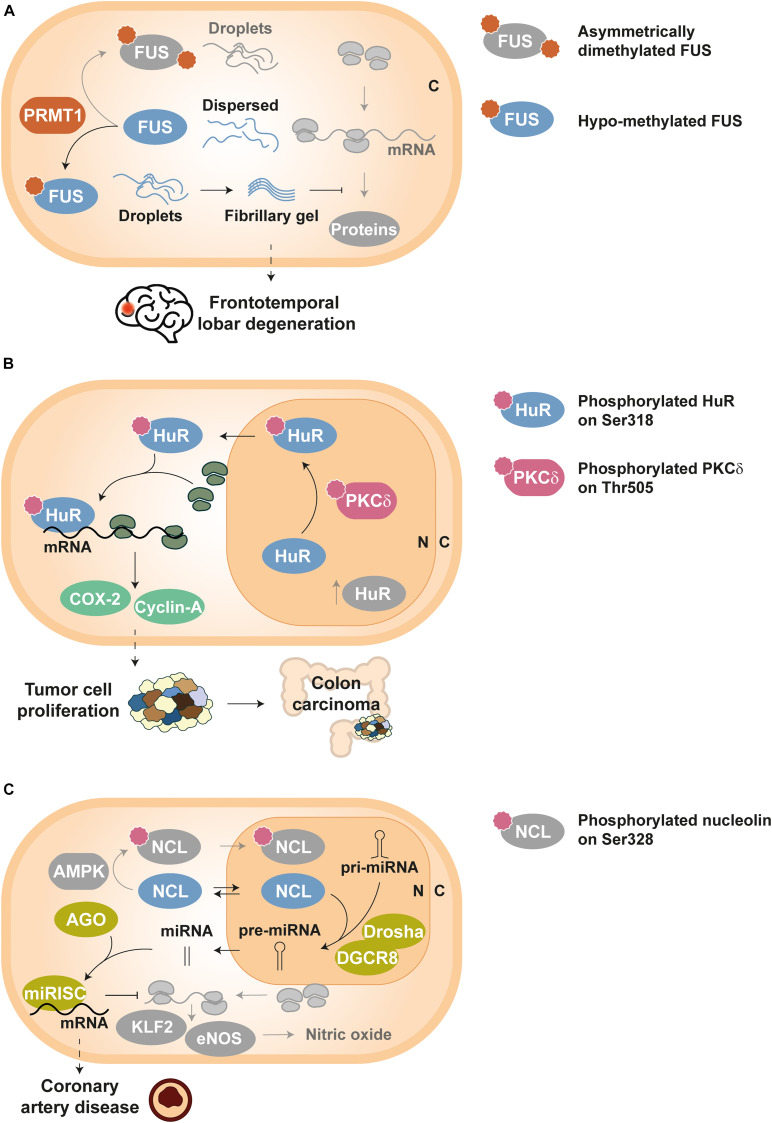
Examples of disease-related dysregulation of PTMs in RBPs. Proteins and components involved in homeostatic pathways are depicted in grayscale, except symbols that stand for PTMs. **(A)** Frontotemporal lobar degeneration (FTLD): FUS molecules can build either droplets or droplets evolving into fibrillary gel state depending on its arginine methylation level, which is controlled by PRMT1 enzymes. Asymmetrically dimethylated FUS yields physiological droplets under homeostatic conditions, whereas hypo-methylated FUS forms highly stable fibrillary gels in FTLD; such fibrillary gels impede normal activity of RNP granules and decrease protein synthesis in neurons (Qamar et al., 2018). **(B)** Tumor cell proliferation: HuR is translocated from the nucleus (N) to the cytoplasm (C) upon PKCδ-dependent phosphorylation at Ser318, thus increasing the stability of tumor related transcripts such as COX-2 and cyclin-A. Elevated levels of Ser318-phosphorylated HuR have been detected in colon carcinoma (Doller et al., 2011). **(C)** Coronary artery disease: unphosphorylated NCL can shuttle between cytoplasm (C) and nucleus (N), and participates in the processing of key pri-miRNAs by the Drosha-DGCR8 complex. Such mature miRNAs associate with AGO proteins to activate the miRISC complex and thus guide the degradation of KLF2 and eNOS mRNAs. As a result, nitric oxide levels in endothelial cells decrease, producing vascular dysfunction (Gongol et al., 2019).

Methylation of RGG motifs by PRMT1 hinders hnRNP A2 *in vitro* self-assembly into condensates by impairing cation–π interactions between arginines and aromatic residues (Ryan et al., 2018). The same mechanism seems to drive FUS demixing, since loss of methylation (Hofweber et al., 2018) and reduced asymmetric dimethylation (Qamar et al., 2018) have been linked to a greater propensity of FUS to form stable aggregates (Figure 2A). In fact, it has been proposed that arginine methylation in RBPs could be a ‘friendly’ PTM, with a protective role against pathological phase transition (Hofweber and Dormann, 2019). Nonetheless, this PTM has also been reported to promote aggregation by enhanced interaction of RGG box-containing RBPs with another phase separating partner (Dammer et al., 2012; Tanikawa et al., 2018) and/or facilitating nuclear export and cytosolic accumulation (Tradewell et al., 2012). Indeed, citrullination, i.e., the conversion of arginine to citrulline, by peptidyl arginine deiminase 4 (PAD4) competes with methylation and reduces the aggregation of ALS-associated proteins (including FUS) and hnRNP A1 (Tanikawa et al., 2018). In principle, both PTMs disrupt the same electrostatic forces that assist the phase transition of RBPs like FUS (St George-Hyslop et al., 2018; Hofweber and Dormann, 2019), although it has also been hypothesized that citrullination inhibits the selective recognition of methylarginines by survival of motor neuron (SMN) proteins and thus prevents co-aggregation (St George-Hyslop et al., 2018; Tanikawa et al., 2018).

Dysfunction of the proteasomal degradation pathway has also been implicated in neurodegenerative disorders (Lukaesko and Meller, 2011). As mentioned before, ubiquitylated TDP-43 is characteristic of inclusions in FTLD and ALS. Increasing evidence associates ubiquitylation of TDP-43 with enhanced self-assembly and aggregation into insoluble droplets (Seyfried et al., 2010; Dammer et al., 2012; Hans et al., 2014), which could be indicative of a deficient turnover regulation of this RBP. Proteomic studies have detected Ub- and SUMO-enriched inclusions, suggesting a possible interplay between both PTMs (Seyfried et al., 2010). Indeed, prior SUMOylation of the ALS-linked FUS P525L mutant was reported to be essential for its destruction via UPS, suppressing its accumulation in SGs. The SUMO-targeted ubiquitin ligase (StUbL) pathway could establish an interdependence relationship between SUMOylation of RBPs in the cell nucleus and dissolution of SGs in the cytosol. A failure in this system was shown to enhance the pathological aggregation of this FUS mutant (Keiten-Schmitz et al., 2020).

### Cancer

Dysregulation of numerous RBPs have been related to cancer and their overexpression can be used as a prognostic marker (Jia et al., 2017; Pereira et al., 2017; Moore et al., 2018; Wolfson et al., 2018; Schuschel et al., 2020). In addition, the aberrant post-translational control of RBPs disrupts their activity and can induce tumor development. For example, HuR phosphorylation at Ser318 by PKCδ promotes its translocation to the cytoplasm, where this RBP stabilizes the mRNAs of cyclooxygenase 2 (COX-2) and cyclin-A (Doller et al., 2007, 2008, 2010, 2011). Since elevated levels of both COX-2 and cyclin-A proteins are associated to abnormal cellular proliferation, Ser318-phosphorylated HuR has been proposed as a tumor marker for colon carcinoma, where cellular concentrations of this phospho-isoform have also been found increased (Figure 2B) (Doller et al., 2011).

Inflammation is a hallmark of cancer (Colotta et al., 2009) and HuR has been implicated in the inflammatory response due to its role in the regulation of the transcripts of many cytokines such as TNF-α, and several chemokines and interleukins (Srikantan and Gorospe, 2012). Recently, PARP1-mediated PARylation at Asp226 was shown to be indispensable for HuR cytosolic translocation (Ke et al., 2017), as well as to promote its oligomerization and to prevent miRISC-mediated decay of HuR transcript targets under inflammatory stimulation (Ke et al., 2021). Moreover, HuR Trp261 was demonstrated as a key residue for mRNA stabilization upon PARylation, indicating that the oligomerization of this RBP is essential for its protective effect (Scheiba et al., 2014; Díaz-Quintana et al., 2015; Pabis et al., 2019; Ke et al., 2021). Intriguingly, it has also been reported that lethal stress induces caspase-7/-3 cleavage of HuR at Asp226, whose proteolytic products promote apoptosis (Mazroui et al., 2008; von Roretz and Gallouzi, 2010). The overexpression of PARP1 observed in several cancers (Sun et al., 2014; Mazzotta et al., 2016; Wang et al., 2017) can produce an uncontrolled PARylation of HuR that may preclude its caspase processing and thus contribute to tumor development (Ke et al., 2021).

Alteration of HuR turnover can elicit the malignant transformation of the cell. For example, the tumor suppressor esophageal cancer-related gene 2 (ECRG2) is upregulated upon DNA damage and promotes HuR ubiquitylation, possibly involving Lys182. As a result, HuR concentration decreases, as does the expression of the X chromosome-linked inhibitor of apoptosis protein (XIAP), whose mRNA is stabilized by this RBP. However, the ECRG2 V30E mutant, found in human lung cancer, cannot reduce HuR levels through the UPS, and enhance cell survival and resistance to chemotherapeutic drugs (Lucchesi et al., 2016). On the other hand, HuR can be NEDDylated at Lys283, Lys313 and Lys326 by the E3 NEDD8-ligase MDM2. These PTMs mobilize HuR to the nucleus and have a protective effect against proteasomal degradation (Embade et al., 2012). Interestingly, increased NEDDylated HuR levels have been detected in liver and colon cancer cells (Embade et al., 2012; Fernández-Ramos and Martínez-Chantar, 2015).

hnRNP K *O*-glycosyl-N-acetylation (*O*-GlcNAc) has been associated to the metastasis of cholangiocarcinoma. This PTM promotes the nuclear translocation of hnRNP K, which acts as a transcription factor of proteins implicated in cellular proliferation, migration and apoptosis inhibition, such as cyclin D1, matrix metalloproteinase 2 and 7 (MMP2/7), and vimentin (Phoomak et al., 2019).

The role of KSRP in miRNA biosynthesis is regulated by the crosstalk between phosphorylation and SUMOylation, and its imbalance could lead to tumorigenesis (Yuan et al., 2017). ATM-mediated phosphorylation of nuclear KSRP at Ser132, Ser274, and Ser670 facilitates its binding to pri-miRNA and boosts generation of mature miRNAs (Zhang et al., 2011), e.g., the let-7 family of tumor suppressors (Trabucchi et al., 2009; Nicastro et al., 2012; Repetto et al., 2012). On the contrary, KSRP SUMOylation at Lys87 by SUMO-1 hinders its interaction with pri-miRNAs and halts processing of let-7 miRNAs by the Drosha-DGCR8 complex, thus preventing their oncogene silencing activity (Yuan et al., 2017).

hnRNP A1 is ubiquitylated by the E3 Ub-ligase TNF receptor-associated factor 6 (TRAF6) (Fang et al., 2017) and then assembles with FUS and the transcription factor c-Jun for the proteasomal degradation of both RBPs (Perrotti et al., 2000). However, the expression of the BCR/ABL oncogene prevents the UPS-mediated destruction of hnRNP A1 and enhances its ability to export mRNAs from the nucleus, possibly via PKCζ-dependent phosphorylation of this RBP. Increased cellular concentrations of hnRNP A1 and altered nucleocytoplasmic traffiking of its target transcripts have been related to BCR/ABL-mediated leukemogenesis (Iervolino et al., 2002; Carrà et al., 2019).

### Other Diseases

The role of RBPs in a broad spectrum of pathologies has been the focus of much research. For example, the correct regulation of HuR sulfhydration has been recently shown to be critical for the physiological function of endothelial cells. Cystathionine γ-lyase (CSE) generates H_2_S, whose ionic form (HS^–^) induces electrophile sulfhydration of HuR at Cys13, blocking its homodimerization. As a consequence, HuR-mediated upregulation of pro-atherogenic E-selectin (CD62E) and cathepsin S (CTSS) mRNAs is impaired. Interestingly, vascular dysfunction and atherosclerosis have been correlated with abnormally high L-cystathionine plasma levels, indicative of CSE inactivation and deficient HuR sulfhydration (Bibli et al., 2019).

The phosphorylation status of NCL has also been proven crucial for vascular endothelial cell homeostasis. NCL Ser328-phosphorylation by AMP-activated protein kinase (AMPK) causes nuclear retention of the RBP and blocks pre-miRNA-93 and pre-miRNA-484 processing. This mechanism allows higher expression of Krüppel-like factor 2 (KLF2) and endothelial nitric oxide synthase (eNOS), key regulators of the vascular function and targets of miRNA-93 and miRNA-484. In contrast, unphosphorylated NCL assists the maturation of the aforementioned miRNAs and the downregulation of KLF2 and eNOS. Interestingly, augmented serum levels of miRNA-93 and miRNA-484 have been correlated to coronary artery disease (Figure 2C) (Gongol et al., 2019).

Ubiquitylation of KSRP has also been associated to metabolic disorders such as atherosclerosis and obesity. The pathological upregulation of the E3 Ub-ligase F-box/WD repeat-containing protein 2 (FBXW2) in macrophages increases KSRP degradation, undermining the normal translational repression of pro-inflammatory factors by this RBP (Wang et al., 2020). On the other hand, ubiquitylated KSRP has also exhibited high activity against picornavirus infection. The C-terminal domain of this RBP is essential for its ubiquitylation, presumably at Lys109, Lys121 and Lys122. Such PTMs give KSRP a competitive advantage for binding to the internal ribosome entry site (IRES) of enterovirus 71 (EV71), inhibiting its cap-independent translation (Kung et al., 2017). However, EV71 eventually induces caspase cleavage of the KSRP C-terminal domain, not only disrupting its anti-infective role, but also transforming this RBP into a positive regulator of viral translation (Chen et al., 2013; Kung et al., 2017).

## Concluding Remarks

The heterogeneous collection of examples presented in this Mini Review illustrates the abundance of factors modulating the impact of PTMs on RBPs. Among them, the crosstalk between PTMs stands out as a key element governing RBPs (Venne et al., 2014; Vu et al., 2018; Huang K. Y. et al., 2019). Different PTMs can exert similar or opposite effects on a given RBP, acting in synergy or interfering with each other, so that certain PTMs assist or exclude the occurrence of additional ones. Furthermore, PTM-specific outputs on RBP biology varies significantly depending on the position and reactivity of the residue affected, the presence or absence of bound partners, and the cellular conditions. Indeed, some relationships can be established between the PTM effect and the role of the modified region. For example, phosphorylation events at or around the RBDs of HuR and hnRNP K usually alter their binding to nucleic acids, while phosphorylation events at nucleocytoplasmic shuttling sequences generally control their subcellular distribution (Grammatikakis et al., 2017; Xu et al., 2019). Similarly, lysine acetylation of FUS in its nuclear localization signal (NLS) restricts its cellular distribution to the cytoplasm, whereas the same PTM in its RRM disrupts its association with RNA (Arenas et al., 2020). Moreover, PTMs at low-complexity IDRs (e.g., arginine methylation of RGG regions) of some phase-separating RBPs such as FUS strongly influence the formation and characteristics of condensates (Hofweber et al., 2018; Qamar et al., 2018; Hofweber and Dormann, 2019), including the irreversible transition into hardened aggregates (Bowden and Dormann, 2016; St George-Hyslop et al., 2018; Fernandopulle et al., 2019; Xue et al., 2020).

Given the well documented connection between many RBPs and their PTMs with a wide variety of diseases, the study of the post-translational regulation of this class of proteins could provide a better understanding of pathophysiological processes. A detailed knowledge of the molecular bases of disease-related dysregulation of PTMs on RBPs hold promise for helping to diagnosis, prognosis and treatment of many severe illnesses by revealing new biomarkers and therapeutic targets. Such a research task will indeed benefit from a multidisciplinary approach that allows investigators to keep pushing the boundaries in this field, through the combination of genomic and proteomic tools, cell-based assays, biophysical techniques and bioinformatic methods (Foshag et al., 2018; Huang R. et al., 2019; Liu and Liu, 2020; Pérez-Mejías et al., 2020; Van Nostrand et al., 2020). Nonetheless, further technological and methodological advances will also be necessary to fully unravel the mechanisms behind the PTM control of RBPs.

## Author Contributions

All authors have made a substantial, direct and intellectual contribution to the work, and approved it for publication.

## Conflict of Interest

The authors declare that the research was conducted in the absence of any commercial or financial relationships that could be construed as a potential conflict of interest.

## Abbreviations

AGO: argonaute protein
ALP: autophagy lysosomal pathway
ALS: amyotrophic lateral sclerosis
AMPK: AMP-activated protein kinase
ARE-RBP: RNA-binding proteins that recognize AU-rich elements
ARE: AU-rich element
ATM: ataxia-telangiectasia mutated
CARM1: coactivator-associated arginine methyltransferase 1
CBP: cAMP-response element binding protein (CREB)-binding protein
CD62E: E-selectin
Chk2: checkpoint kinase 2
Clk1: Cdc-like kinase 1
COX-2: cyclooxygenase 2
CSE: cystathionine γ-lyase
CTSS: cathepsin S
CUREs: CU-rich elements
DDR: DNA damage response
DGCR8: DiGeorge syndrome critical region 8
DICE: differentiation control element
ECRG2: esophageal cancer-related gene 2
eNOS: endothelial nitric oxide synthase
ERK: extracellular signal-regulated kinase
EV71: enterovirus 71
FASTK: Fas-activated serine/threonine kinase
FBXW2: F-box/WD repeat-containing protein 2
FTLD: fronto-temporal lobar degeneration
FUS: fused in sarcoma
FXR2P: fragile X-related protein 2
GM-CSF: granulocyte-macrophage colony-stimulating factor
GSK3 β: glycogen synthase kinase 3 β
G3BP1: Ras-GAP SH3 domain-binding protein 1
HDM2/MDM2: human/mouse double minute 2
hnRNP A1/A2/K: heterogeneous nuclear ribonucleoprotein A1 A2 and K
HNS: HuR nucleocytoplasmic shuttling sequence
Hsp27: heat shock protein 27
HuR: human antigen R
IDR: intrinsically disordered region
IKK α: I κ B kinase α
IRES: internal ribosome entry site
JAK3: Janus kinase 3
KH: K homology
KI: K interactive region
KLF2: Krüppel-like factor 2
KNS: K nuclear shuttling domain
KSRP: KH-type splicing regulatory protein
LCR: low-complexity region
LLPS: liquid–liquid phase separation
MAPK: mitogen-activated protein kinase
MAPKAPK-2/3: MAPK-activated protein kinase 2 and 3
mFas: membrane-bound Fas
miRISC: miRNA-induced silencing complex
miRNA: micro-RNA
MK2/3: MAPK-activated protein kinase 2 and 3
MLO: membrane-less organelle
MMP2/7: matrix metalloproteinase 2 and 7
mRNP: messenger ribonucleoprotein particle
NCL: nucleolin
NEDD8: neural precursor cell expressed developmentally downregulated 8
NLS: nuclear localization signal
*O*-GlcNAc: *O*-glycosyl -*N*-acetylation
PABP: poly(A)-binding protein
PAD4: peptidyl arginine deiminase 4
PAR: poly(ADP-ribose)
PARP-1: PAR polymerase 1
Pc2: polycomb 2 protein
Pin1: protein interacting with NIMA (never in mitosis A)-1
PKA: protein kinase A
PKC α/δ/ζ: protein kinase C α δ and ζ
PLD: prion-like domain
pre-miRNA: precursor miRNA
pri-miRNA: primary miRNA
PRMT1: protein arginine N-methyltransferase 1
PTH: parathyroid hormone
PTM: post-translational modification
r15-LOX: erythroid-15-lipoxygenase
RBD: RNA-binding domain
RBP: RNA-binding protein
RGG/RG: arginine/glycine-rich
RRM: RNA recognition motif
sFas: soluble Fas
SGs: stress granules
SIRT1: Sirtuin 1
SMN: survival of motor neuron
snRNP U1: small nuclear ribonucleoprotein particle U1
SR: serine/arginine-rich
SRPK1/2: SR protein kinase 1 and 2
SRSF1/3: SR splicing factor 1 and 3
StUbL: SUMO-targeted ubiquitin ligase
SUMO: small ubiquitin-like modifier
TDP-43: TAR DNA-binding protein of 43 kDa
TIA-1: T-cell intracellular antigen 1
TIAR: TIA-1-related
TIAR: TIA-1-related protein
TRAF6: TNF receptor-associated factor 6
TRN: transportin
TTP: tristetraprolin
Ub: ubiquitin
UBXD8: ubiquitin regulatory X domain-containing protein 8
UCP2: uncoupling protein-2
UPS: ubiquitin-proteasome system
UTR: untranslated region
XIAP: X chromosome-linked inhibitor of apoptosis protein
β-TrCP 1: β-transducin repeat-containing protein 1.
