# Supplementary material for: Post-translational Control of RNA-Binding Proteins and Disease-Related Dysregulation
Source: Front Mol Biosci. 2021 Apr 27;8:658852. doi: 10.3389/fmolb.2021.658852 (PMC8111222; doi:10.3389/fmolb.2021.658852)
Supplement: Supplementary file 1 [file Table_1.pdf]

<table>
<tr><th>PTM</th><th>Unmodified Residue</th><th>Modified Residue</th></tr>
<tr><td>Acetylation</td><td>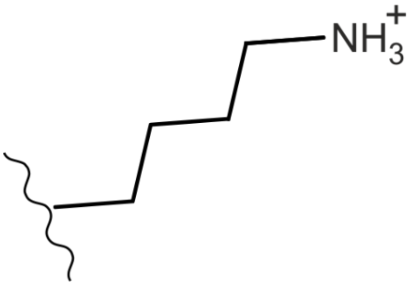

Lysine</td><td>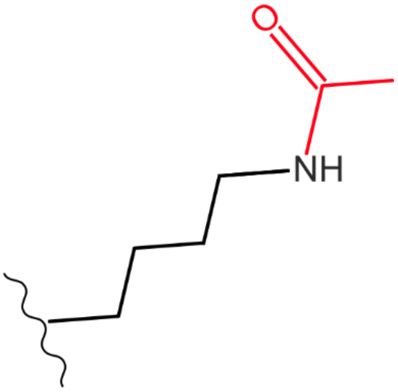

Acetyllysine</td></tr>
<tr><td>Citrullination</td><td>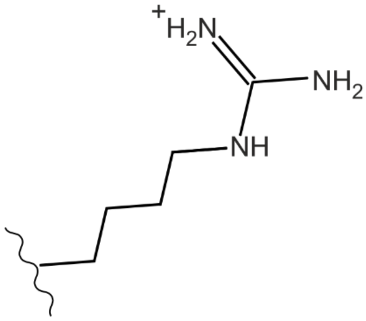

Arginine</td><td>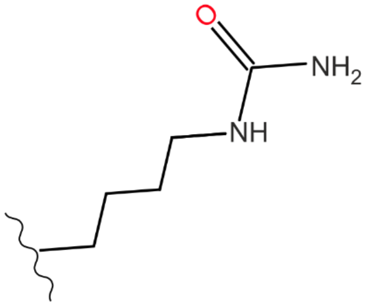

Citrulline</td></tr>
<tr><td rowspan="2">Glycosylation (O-glycosyl-N-acetylation)</td><td>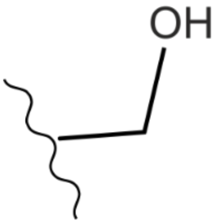

Serine</td><td>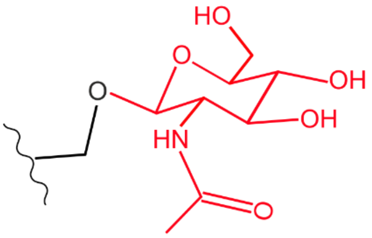

O-GlcNAcylated serine</td></tr>
<tr><td>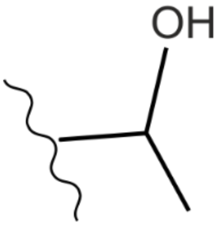

Threonine</td><td>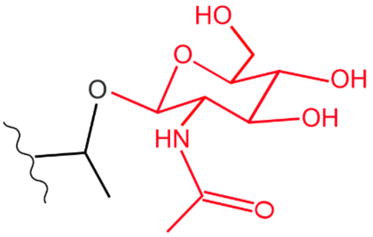

O-GlcNAcylated threonine</td></tr>
</table>

PTM	Unmodified Residue	Modified Residue
Hydroxylation	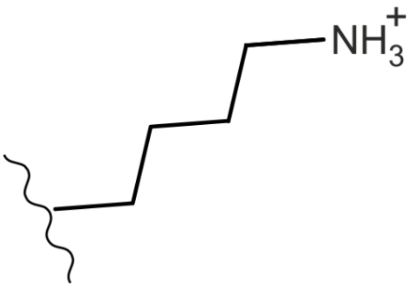<br>Lysine	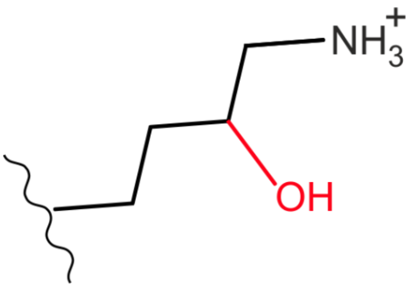<br>Hydroxylysine
	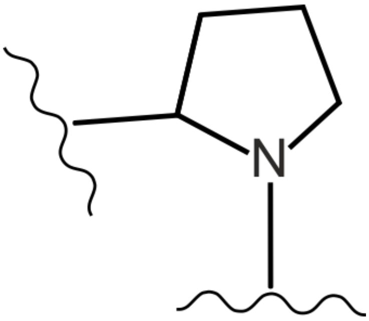<br>Proline	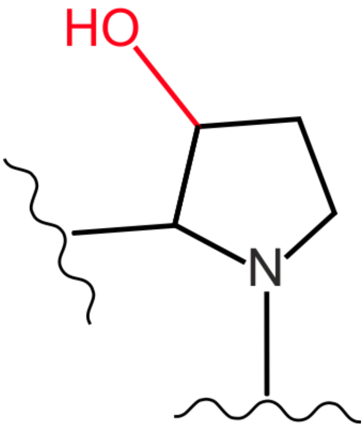<br>Hydroxyproline
Isomerization	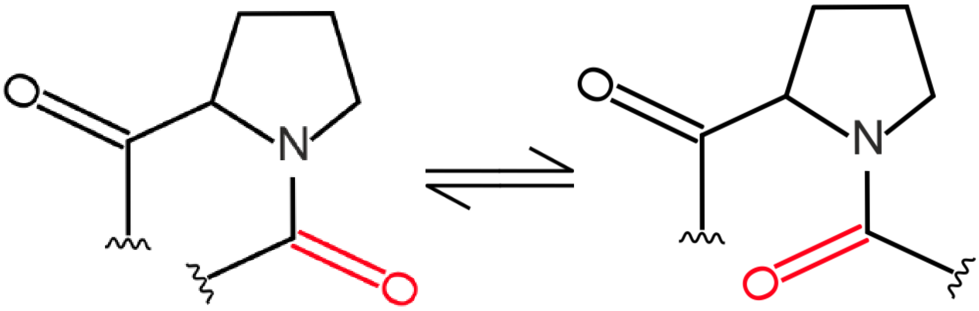<br>*cis* Proline *trans* Proline	

<table>
<tr><th>PTM</th><th>Unmodified Residue</th><th>Modified Residue</th></tr>
<tr><td rowspan="4">Methylation</td><td rowspan="3">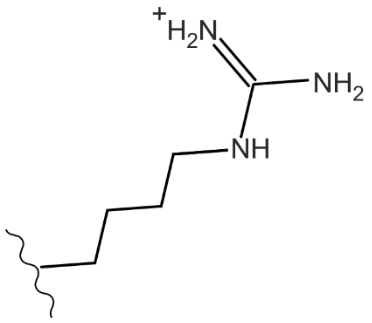
Arginine</td><td>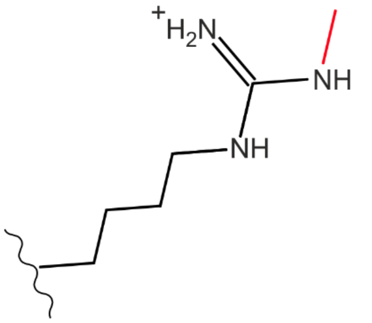
N^{G}-monomethylarginine</td></tr>
<tr><td>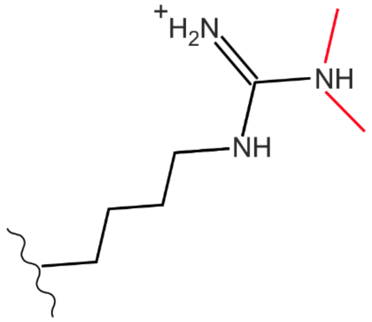
Asymmetric dimethylarginine</td></tr>
<tr><td>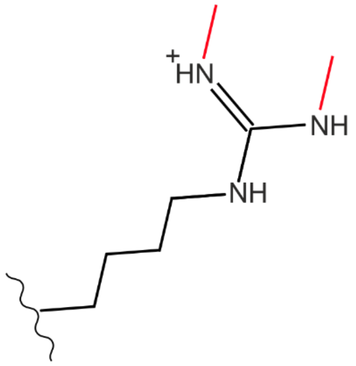
Symmetric dimethylarginine</td></tr>
<tr><td>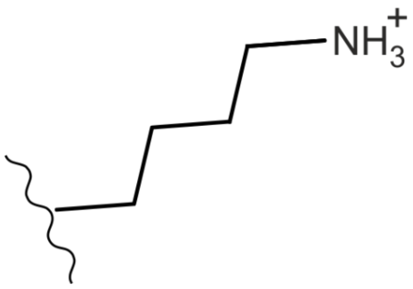
Lysine</td><td>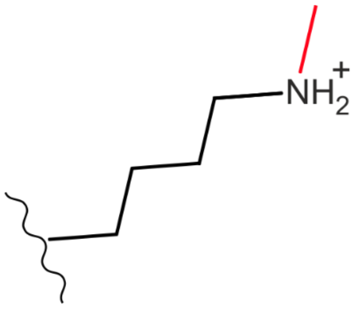
Monomethyllysine</td></tr>
</table>

PTM	Unmodified Residue	Modified Residue
Methylation	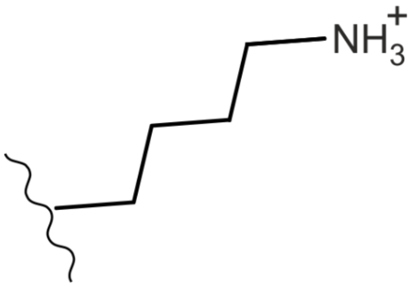<br>Lysine	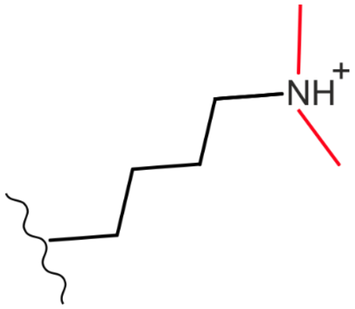<br>Dimethyllysine
		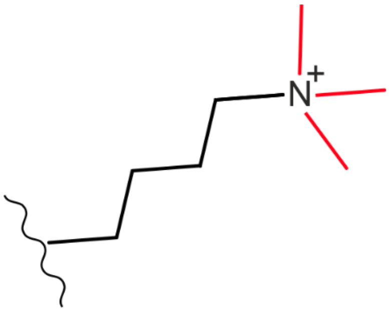<br>Trimethyllysine
Myristoylation	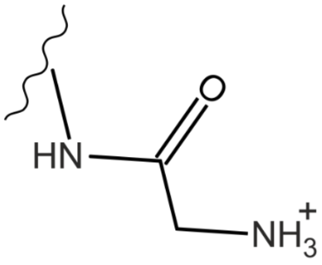<br>N-terminal glycine	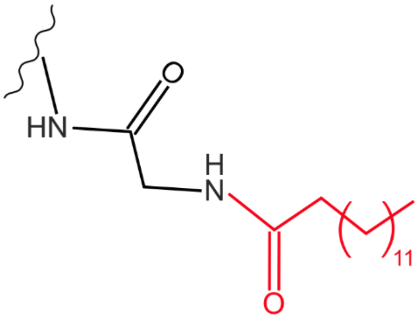<br>Myristoylated glycine
PARylation (polyADP-ribosylation)	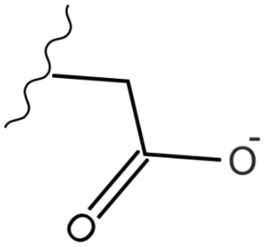<br>Aspartate	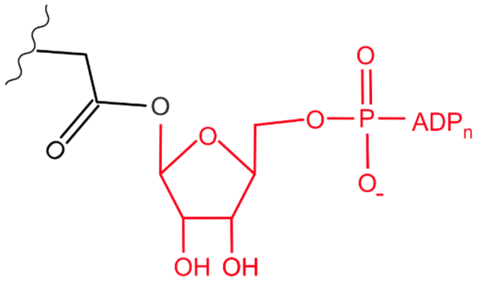<br>PARylated aspartate

PTM	Unmodified Residue	Modified Residue
PARylation (polyADP-ribosylation)	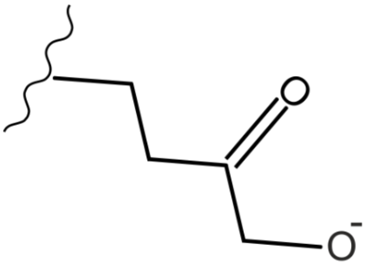 Glutamate	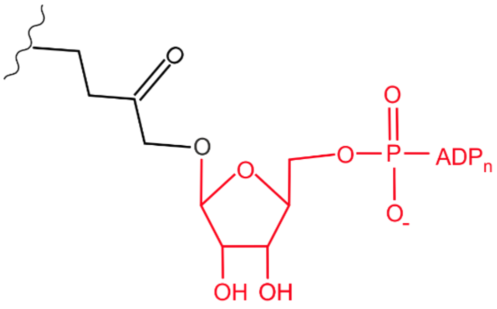 PARylated glutamate
	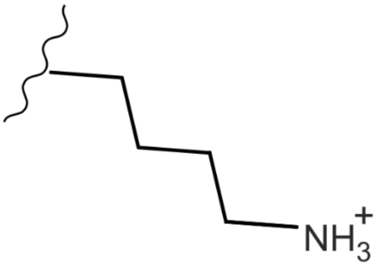 Lysine	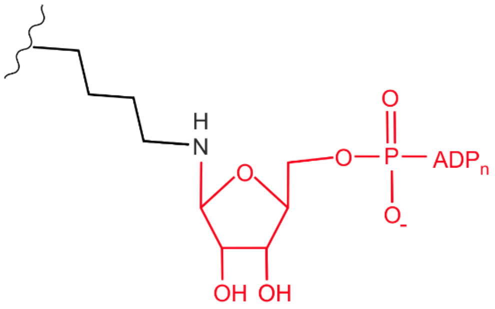 PARylated lysine
Phosphorylation	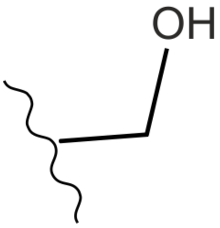 Serine	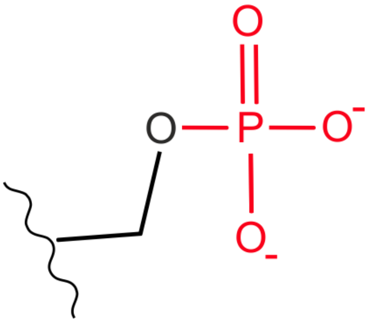 Phosphoserine
	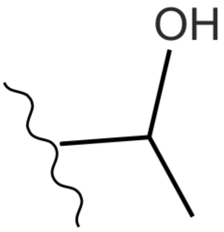 Threonine	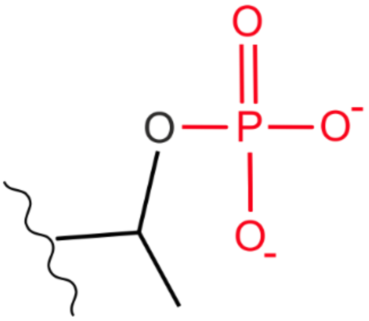 Phosphothreonine

PTM	Unmodified Residue	Modified Residue
Phosphorylation	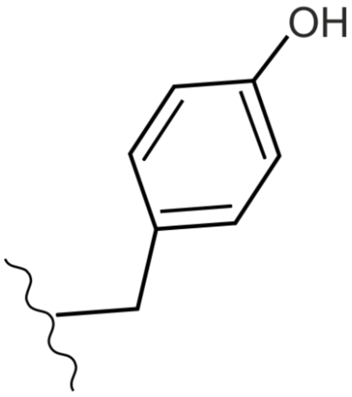 Tyrosine	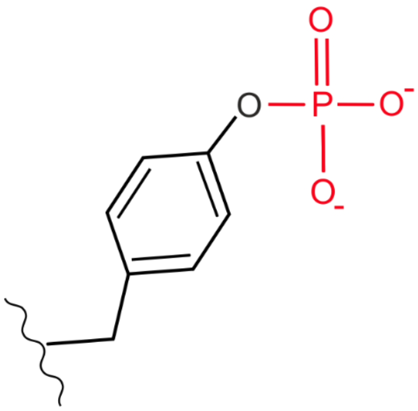 Phosphotyrosine
Proteolysis (caspase cleavage)	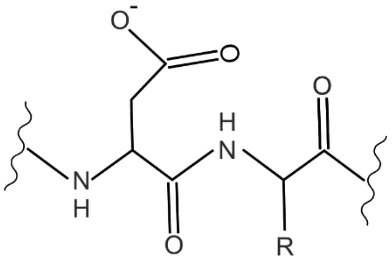 Aspartate-amino acid	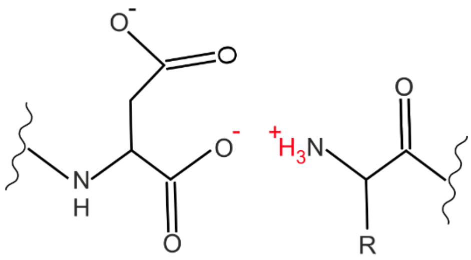 Cleavage after aspartate
S-sulfhydration	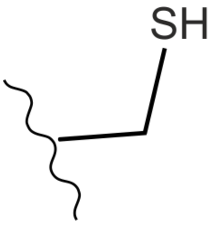 Cysteine	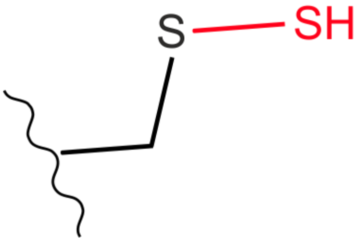 S-sulfhydrated cysteine
Ubiquitin (Ub) and Ub-like conjugation	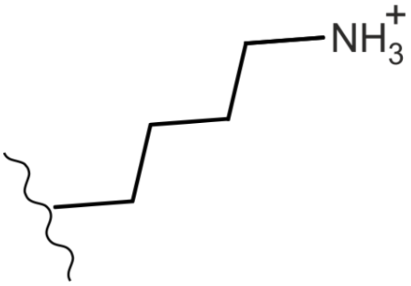 Lysine	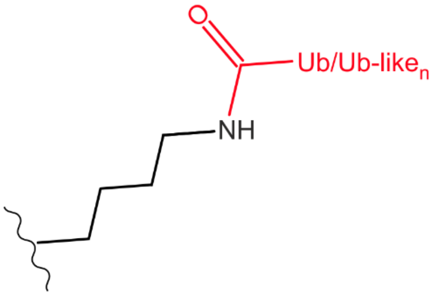 Ub-/SUMO-/NEDDylated lysine

Table S1. List of PTMs described for RBPs. Chemical modifications are highlighted in red.

AUF-1	AU-rich binding factor 1
FUS	Fused in sarcoma
FXR2P	Fragile X-related protein 2
G3BP1	Ras-GAP SH3 domain-binding protein 1
hnRNP A1/A2/K	Heterogeneous nuclear ribonucleoprotein A1, A2 and K
Hsp27	Heat shock protein 27
HuR	Human antigen R
KSRP	KH-type splicing regulatory protein
NCL	Nucleolin
SRSF1/3	Serine/arginine-rich splicing factor 1 and 3
TDP-43	TAR DNA-binding protein of 43 KDa
TIA-1/TIAR	T-cell intracellular antigen 1 and TIA-1-related protein
TTP	Tristetraprolin

Table S2. List of RBPs referenced in this Mini Review by alphabetical order.
